# Atypical phenotypic aspects of autoimmune thyroid disorders in young patients with Turner syndrome

**DOI:** 10.1186/s13052-018-0447-3

**Published:** 2018-01-17

**Authors:** Tommaso Aversa, Romina Gallizzi, Giuseppina Salzano, Giuseppina Zirilli, Filippo De Luca, Mariella Valenzise

**Affiliations:** Department of Human Pathology in Adulthood and Childhood, University of Messina, Via Consolare Valeria, 98124 Messina, Italy

**Keywords:** Graves’ disease, Hashimoto’s thyroiditis, Metamorphic autoimmunity, Natural history, Thyroid status

## Abstract

Aim of this commentary is to analyze the current views about the phenotypic features of Hashimoto’s thyroiditis (HT) and Graves’ disease (GD) in Turner syndrome (TS) girls, in terms of epidemiology, clinical and biochemical presentation, long-term course and metamorphic autoimmunity evolution. In TS GD course is not atypical, whereas HT course is characterized by both a mild presenting picture and a severe long-term evolution of thyroid function tests. Furthermore, TS girls seem to have an increased risk of switching over time from HT to GD. On the light of these findings, it may be concluded that TS girls with HT need a careful monitoring of thyroid status over time.

Conclusions: 1) In children the association with TS is able to condition a peculiar phenotypic expression of HT in terms of epidemiology, presentation course and long-term metamorphic autoimmunity; 2) by contrast, children with TS do not exhibit an atypical clinical and biochemical course of GD, but only a significantly higher prevalence of this disease.

## Background

Turner syndrome (TS) is one of the most common chromosomopathies, with a reported prevalence of 1:2500 live-born females [1]. Individuals with TS have an increased risk for celiac disease and autoimmune thyroid disorders (AITDs), but also for type 1 diabetes, vitiligo and juvenile idiopathic arthritis [2, 3]. Hashimoto’s thyroiditis (HT) is, by far, the most common autoimmune disease in TS girls [2, 4], whilst the association between this syndrome and Graves’ disease (GD) has been reported to be significantly more infrequent [5–7].

The reasons for predilection of the thyroid gland as a target of autoimmunity in patients with TS have not been clearly identified to date [8], but this predilection might be simply explained, at least partially, on the basis of the close association between thyroid autoimmunity and female gender [5].

A very interesting aspect, which emerges from the analysis of the most recent reports on the relationships between TS and AITDs, is that the association with TS seems to be able to affect the phenotypic expression of AITDs in pediatric age, with significant repercussions on their presentation and long-term course.

Aim of this commentary is to analyze the current views about the phenotypic features of TS-related AITDs in terms of epidemiology, clinical and biochemical presentation, long-term course and metamorphic autoimmunity evolution.

## Epidemiology and pathophysiology of AITDs in TS girls

If compared with girls without TS, young patients with TS are distinctly more incline to develop AITDs. In fact, the prevalence of both HT and GD was reported to be significantly higher in TS girls than in the pediatric general population: respectively 10–20% [2, 5, 8, 9] vs around 1.2% [10] for HT and 1.7–3% [5, 6] vs 1.07‰ [11] for GD.

The increased susceptibility of TS girls to AITDs might be due to haploinsufficiency of the genes in the pseudoautosomal region of X-chromosome [3, 12], which are known to be possibly involved in the immunoregulation process [12]. Another mechanism that has been postulated to be, at least partially, responsible for the increased susceptibility of TS girls to AITDs is an up-regulation of proinflammatory cytokines [13]. Finally, a decrease in the CD4: CD8 lymphocytic ratio, that has been observed in TS children, may play a key-role in conditioning the predisposition of these girls to autoimmunity.

Whatever the mechanism of the enhanced susceptibility of TS girls to AITDs, it may be argued, however, that these patients are, per se, more exposed to this risk, irrespective of familial predisposition, or association with other autoimmune disorders. In fact, it was reported that the frequency of family HT antecedents in a cohort of TS patients with HT may be significantly lower than that detected on a control pediatric population and that the association with other autoimmune extra-thyroidal disorders may not be more common than in controls [14].

Finally, it has to be emphasized that the majority of studies did not report any significant relationships between AITDs and a specific karyotype in TS individuals [4, 7, 15], which may suggest that these patients are more prone to develop AITDs irrespectively of karyotype. Nevertheless, other authors had previously reported that AITDs may be more common in TS patients with X-isochromosome karyotype [5, 16]. Therefore, this last point cannot be considered completely clarified.

## Presentation and evolution of GD

The available studies aiming to investigate the course of GD in TS patients are sparse and based on scarce populations [5–8, 12]. According to the results of the most recent one, it is possible to infer that GD in TS individuals presents later than in the pediatric general population [7], which is not surprising considering that the frequency of TS-related AITDs has been reported to increase with age and to double from the first to the third decade of life [2].

The clinical picture at presentation is not atypical, being characterized by the same manifestations that are generally observed in GD children without TS: goiter, tachycardia, weight loss and other hyperthyroid symptoms [7]. Nevertheless, ophthalmopathy has been reported to be initially very infrequent and FT4 serum levels, at the time of diagnosis, have been found to be lower than those detected in GD children without TS [7]. Both these findings seem to indicate that diagnosis of hyperthyroidism in TS may be probably assessed in an earlier phase, since these girls, at the time of GD onset, are already under special control and have regular consultations with endocrinologists [7].

Also the subsequent clinical course of GD does not seem to be different in the patients with TS than in those without TS [7]. In fact, the initial remission rates after the start of methimazole treatment, the relapse rates after the first methimazole treatment cycle withdrawal, the prolonged remission rates after a 2-year withdrawal of pharmacological therapy and even definitive remission rates were found to be very similar in the two groups with or without TS [7]. It has also to be underlined that the initial methimazole doses and those needed to maintain euthyroidism were not different in the two groups. Finally, even the rates of girls who need a non-pharmacological treatment do not seem to be significantly higher in GD girls with TS [7].

## Presentation and evolution of HT

**Fig. 1:**
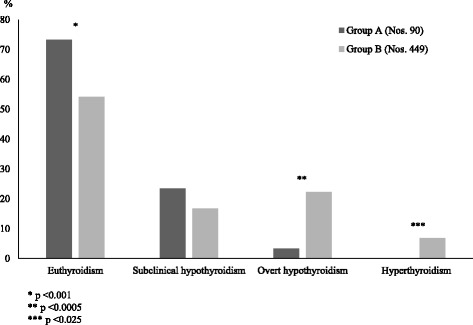
Prevalences (%) of the main biochemical pictures of thyroid function detected, at diagnosis of Hashimoto’s thyroiditis (HT), in two groups of HT girls with (Group A) or without (Group B) Turner syndrome (according to the results of Reference [14] study)

According to the results of the first study aiming to compare the presentation biochemical patterns of HT in two groups of children with or without TS [14], HT presents with a less severe hormonal picture in TS girls. In fact, the girls with TS exhibited, at the time of HT diagnosis, a significantly higher prevalence of euthyroidism and a lower prevalence of both overt hypothyroidism and hyperthyroidism, whereas the prevalence of subclinical hypothyroidism (SH) was very similar in the two cohorts (Fig. 1). Moreover, at entry, TS patients showed lower median TSH levels, when compared to those without TS [14].

The less severe biochemical pattern of thyroid function that was initially found in TS series might be explained on the light of a less aggressive autoimmune pattern, as also suggested by the significantly lower thyroid autoantibody levels which were detected, at HT diagnosis, in TS girls [14]. Another possible explanation is that several pediatricians are aware that TS girls have a more elevated risk of developing AITDs and, therefore, may be on the alert, even in presence of minimal symptoms.

**Fig. 2:**
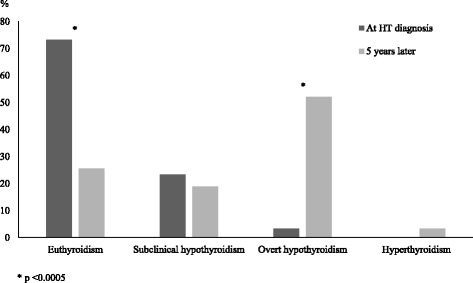
Prevalences (%) of the main biochemical pictures of thyroid function detected in 90 girls with Turner syndrome and associated Hashimoto’s thyroiditis (HT), at the time of HT diagnosis and 5 years later (according to the results of Reference [14] study)

By contrast, the evolution of thyroid status over time in girls with TS is characterized by a severe deterioration of thyroid tests, during a 5-year follow-up, in an elevated percentage of patients (Fig. 2).

Therefore, it is possible to argue that the association with TS is able to impair the long-term prognosis of thyroid function in girls with HT. Such a negative effect is more evident in the TS patients presenting with SH [14, 17], but may be observed even in initially euthyroid girls [18]. Furthermore, this effect is not necessarily linked with a specific karyotype [18].

## Metamorphic thyroid autoimmunity

HT and GD are caused by two distinct and separate paradigms [16] and were regarded for a long time as two different entities. Nevertheless, more recent views have taken into consideration the hypothesis that these conditions might be two ends of the AITD spectrum [19]. In fact, it has been sporadically reported that GD and HT may follow one another in the same individuals [19], due to a sequential phenotypic conversion from HT to GD [20–22], or vice versa [23]. The most common metamorphic scenario concerns the progression from GD into HT, whilst the metamorphosis from HT into GD seems to be less common [19, 20].

Among the variables that have been postulated to be able to affect the shifting process from HT into GD, a predisposing role has been recently ascribed to the association with either TS or Down’s syndrome (DS) [19, 24]. In fact, patients with either TS or DS and associated HT were found to be at higher risk of progressing toward GD [24].

## Follow-up and management of TS girls with AITDs

GD generally presents with a severe clinical and biochemical picture in both patients with or without TS and a therapeutic intervention is, therefore, needed in all cases. By contrast, HT presentation in children and adolescents without TS may be very various [25, 26] and treatment has to be necessarily and immediately started only in the cases presenting with overt hypothyroidism [27, 28].

In the girls with TS, the natural course of HT is more severe than in those without TS, being frequently characterized by a significant deterioration of thyroid status over time [14, 17–19]. Therefore, it may be inferred that these girls should require continued monitoring of thyroid function from 4 years of age onward [29], as also suggested by the TS Study Group Consensus in 2007 [30]. According to the statements of the last and very recent guidelines of the International TS Study Group Consensus, it is recommended to screen for thyroid dysfunctions all the girls at diagnosis of TS. Subsequently, all these patients should undergo annual measurements of FT4 and TSH from early childhood and throughout life-span [31].

Anti-thyroid autoantibody measurement is recommended at diagnosis of HT or at the first detection of thyroid enlargement. Thyroid ultrasonography is not always necessary for HT diagnosis [31].

## Conclusions

1) In children, the association with TS is able to condition a peculiar phenotypic expression of HT in terms of epidemiology, presentation course and long-term metamorphic autoimmunity; 2) by contrast, children with TS do not exhibit an atypical clinical and biochemical course of GD, but only a significantly higher prevalence of this disease.

## Abbreviations

AITDs: Autoimmune thyroid disorders
DS: Down syndrome
GD: Graves’ disease
HT: Hashimoto’s thyroiditis
SH: Subclinical hypothyroidism
TS: Turner syndrome
